# Medium-fidelity simulation in clinical readiness: a phenomenological study of student midwives concerning teamwork

**DOI:** 10.1186/s12912-018-0303-1

**Published:** 2018-07-27

**Authors:** Zukiswa Brenda Ntlokonkulu, Ntombana Mc’deline Rala, Daniel Ter Goon

**Affiliations:** Department of Nursing Science, Faculty of Health Sciences, University of Fort Hare, East London, South Africa

**Keywords:** Medium-fidelity simulation, Teamwork, Obstetric emergency, Interpretative phenomenological analysis

## Abstract

**Background:**

Teamwork during obstetric emergency ensures good outcomes for both the woman and her baby. Effective teams are characterised by mutual respect, support, and cooperation among team members.

**Methods:**

This qualitative, interpretive, phenomenological analysis study was conducted on a purposive sample of five, fourth-year Bachelor of Nursing Science student midwives at the University of Fort Hare (UFH). In-depth semi-structured interviews were conducted. Data analysis applied the interpretative phenomenological analysis method.

**Results:**

Superordinate theme demonstrated teamwork elicited four clustered themes namely delegation of duties, the importance of teamwork, team support, and confident team leader. The participants recognised that there should be a team leader who is capable of delegating duties to other team members in the management of an obstetric emergency, Participants were confident not only to assign duties but to be kept updated of the intervention. They expressed the need to work collaboratively as a team to achieve the desired goal of providing quality care to the woman. The participants maintained that the team must be supportive and be able to help in decision making during simulation of an obstetric emergency. A sense of mutual respect is echoed by some participants in the process of caring for the woman. Some participants were confident at being team leaders and could see themselves as leaders in the real-life clinical situation.

**Conclusion:**

The participants acknowledge the importance of teamwork in resolving obstetric emergencies. The importance of delegating duties to other team members, providing updated progress report ensures better outcomes for the woman.

## Background

Simulation has been used in nursing education to teach both technical and non-technical skills such as teamwork. It is evident that simulation based on teamwork prepares healthcare personnel to manage obstetric emergencies in a practical setting [1]. In midwifery, an obstetric emergency requires teamwork for better outcomes for both the woman and her baby. Apart from the lecture method, which appears to be less attractive in the modern era in terms of applicability and skills acquisition, the experiential learning involving simulation can be used in healthcare team training [2]. Moreover, simulation is ideal for team training as it is hands-on and can encourage collaboration among participants [3]. Teamwork is imperative in any kind of emergency. During obstetric emergencies, staff members need to consolidate their learning to function as a team for better outcomes such as the safety of the woman and her baby.

Globally, there has been a commendable concern for patient safety in health care settings [4, 5]. The World Health Organisation (WHO) has stressed the importance of educating health-care professionals on the principles and concepts of patient safety [6]. Countries such as Australia and England have made patient safety a priority in government agenda [7, 8]. In South Africa, the Department of Health has made patient safety one of its priorities through the implementation of the strategic plan for nurse education, training and practice [9].

The institutions of health care professional training have committed themselves to provide training programmes that are designed for hospital-specific risks. Patient safety has always been part of the nursing curriculum for years but its lack of transferability in practice has been well documented [10]. Cockerham [11] states that patient safety is the ultimate attribute that a newly qualified nurse should possess. Debourgh and Prion [10] assert that the clinical experience of a student in team performances and patient safety is often limited by the student role and scope of practice. However, due to the lack of opportunities for the students to practice during real clinical emergency simulation provides an ideal environment where team processes and behaviour can be learned without putting a patient at risk [12, 13]. The growing interest in simulated learning over the years is born out of concern for patient safety [14, 15]. Patient safety during simulation is further reinforced during the debriefing session allowing the student to assimilate learning [16].

There is a dearth of research on nursing teamwork, despite evidence that many errors committed by nurses are partly due to poor teamwork. Deering et al. [17] assert that complications in health care are not attributed to the individual but to team performance failure. A focus on team performance and training started in the aviation discipline, then proceeded to the army to improve safety [17]. Several studies have shown that strategies to enhance teamwork in health care have been adapted from these high-risk professions [2–4]. Training programmes in obstetric emergencies, such as Managing Obstetrical Emergencies and Trauma (MOET) [18], Multidisciplinary Obstetric-Simulated Emergency Scenarios (MOSES) [19], Practical Obstetric Multi-Professional Training (PROMPT) [20] are used for both clinical and non-clinical skills such as teamwork and woman safety. These training programmes have been adapted to meet the training needs of healthcare workers in developing countries such as South Africa and Zimbabwe [21]. Incorporating PROMPT in the training programme resulted in increased staff confidence in the management of emergencies, improved teamwork and inter-professional relations [20].

In midwifery, midwives work as an interdisciplinary and multi-disciplinary team. This is evident in an obstetric emergency which requires the presence of a multi-disciplinary team; who have different pieces of training and sometimes may not understand the scope of practice of each discipline. Whilst there are studies in obstetrics focusing on interdisciplinary and multi-disciplinary teams during obstetric emergency simulation [19, 22, 23]. There is a paucity of literature on obstetric emergency team simulation tailored mainly for midwives. Medium-fidelity simulation can be of benefit to training midwives in the management of obstetric emergencies.

Medium fidelity simulation (MFS) is the use of manikins or task trainers that offer breath sound, heart sounds, bowel sound or simulated blood but lack the authenticity of a realistic environment. Medium-fidelity simulation is a cost-effective method to train student midwives in both technical and non-technical skills such as teamwork and leadership obstetric. Medium-fidelity simulation creates a realistic environment where students can learn to manage obstetric emergencies as part of a team. Despite the availability of MFS at UFH, its benefit on the clinical readiness of student midwives is not known. Would student midwives be able to acquire attributes that are needed in team performance during an obstetric emergency?

### Aim

The aim of the study was to explore, describe and analyse the views of student midwives concerning teamwork during medium-fidelity obstetric emergency simulation.

### Research design

An interpretative phenomenological analysis (IPA) approach was used to explore, describe and analyse the lived experiences of student midwives with regards to teamwork during a medium fidelity obstetric emergency simulation. The student midwives’ individual experiences of the post-partum haemorrhage (PPH) simulation demonstrated the diverse experiences of the same phenomenon which are the reflective, interpretative and idiographic premises of IPA [24].

### Population

The target population was a fourth-year Bachelor of Nursing Science student midwives at the University of Fort Hare. The inclusion criteria were the fourth-year student midwives who had passed the first semester’s midwifery module. A purposive sampling method was used to select five fourth-year student midwives who were the team leaders during the management of postpartum haemorrhage (PPH) using MFS. Fourth-year student midwives were selected because, at the University of Fort Hare, obstetric emergencies are taught in the second year of midwifery. Usually, high-risk midwifery involves emergencies that require teamwork. Ethical approval of the study was obtained from the University of Fort Hare Ethics Committee. Permission to conduct the study was granted by the Head of Department of Nursing Sciences, University of Fort Hare.

### Trustworthiness

Trustworthiness of this study was ensured by applying the principles of trustworthiness, namely transferability, credibility, confirmability, and dependability as outlined by Guba [25]. Transferability of the study was ensured by keeping both hard and soft copies of the research steps taken that can be accessed on request. Both the soft and hard copy of the data will be made available online at the university repository. The credibility of the findings was determined by taking the research transcripts to a co-coder for data validation. Confirmability was attained by ensuring that there was enough data to support the findings and conclusions. Dependability was ensured by narrating a detailed description of the research design and the steps taken in data collection.

### Ethical considerations

Ethical approval of the study was obtained from the University of Fort Hare Ethics Committee. Permission to conduct the study was granted by the Head of Department of Nursing Sciences, University of Fort Hare. Three fundamental ethical principles were applied, namely; the principle of respect for the person, beneficence, and justice [26].

### The principle of respect for the person

The student midwives were informed of the right to refuse to participate and to withdraw from the study at any given time without any prejudice or penalty. The nature and aim of the study were explained to the participants, prior to data collection.

### Principle of beneficence

The principle of beneficence refers to the individual’s right to protection from harm. This study was non-invasive, and the student midwives were informed that the simulation was not for assessment reasons but for exploring their experiences of the simulation.

### Principle of justice

The principle of justice refers to how the researcher comes to choose the study population. The student midwives’ right to anonymity was maintained. Before the beginning of each interview, permission was sought from each student midwife to use a pseudonym during the interview. Participants were made to understand their right to the privacy of any information provided, and their consent was sought before the use of a tape recorder. Prior to the interview, each student midwife completed an informed consent form with a clear explanation. The participants had the right to ask any question(s) during and after interviews.

## Methods

An Essential Steps in Management of Obstetric Emergency (ESMOE) post-partum haemorrhage video which was sent on-line through Blackboard to all fourth-year student midwives in order to demonstrate the process. Management of PPH is one of the contents that are taught in the midwifery abnormal pregnancy, labour and puerperium module. The student midwives had an opportunity to watch the video repeatedly in order to thoroughly comprehend the demonstrated skill. The day before the simulation the six bedded simulation laboratory was prepared for the PPH simulation. Only four-bed spaces were used for the simulation. The advanced OB Susie a Gaumard with an audible foetal and maternal pulse was filled with simulated blood and urine and was positioned in the centre of the bed. Four large dressing trolleys were prepared with all the equipment needed for the management of PPH. Each bed space had an oxygen outlet with an oxygen mask and two drip stands and a blood pressure machine and relevant ward documentation. On the day of the simulation, the standardised patient (SP) was given a scenario. The Nursing Science Department recruit members of the community to pretend to be SPs for some of the undergraduate practical program. The SPs were provided with a scenario. Critical points of the scenario were explained to the SPs such as when to call for the nurse, when to pass out and when to regain consciousness, what question to ask. The SPs clad in a hospital gown was positioned above the OB Susie torso to create a hybrid. The hybrid was draped with green towels. On the day of the simulation, the students were given the simulation scenario to read prior to the simulation. The ESMOE video was played for the group the second time before the demonstration of the simulation. The students decided among themselves which roles to play during the simulation.

## Data collection

Fourth-year midwifery students were recruited by the first author (ZN) during their theory block. The 4th year students were approached in class after a lecture. The aim and nature of the study were explained to the students. ZN is the simulation laboratory manager and had no prior relationship with the students. A semi-structured interview guide was used with nine questions. Open-ended questions were used in order to allow the student midwives the opportunity to explore their lived experiences of an obstetric emergency using medium-fidelity simulation. Prompts during the interview centred on the students’ experiences of obstetric emergencies using medium-fidelity simulation, for example, how did the participant feel being the team leader? How was the communication between team members? Individual interviews were conducted at the University of Fort Hare’s simulation laboratory by the first author (ZN) over a period of 1 month, each interview session lasted between 26 min and 44 min. A Samsung smartphone was used to record the interviews, and a notepad was used to make notes of gestures such as smiles or other facial expressions. After each interview, the recorded interview was transferred onto a laptop and a file was opened for the interviewee, identified by a pseudonym. The interviews were transcribed verbatim as Word documents by the first author.

### Data analysis

Data were analysed by the first author following the six steps suggested by Smith et al. [24] namely reading and re-reading, initial noting, developing emergent themes, searching for connections across the emergent themes, moving to the next case and looking for patterns across cases. The transcript was read through twice. The first reading was done whilst listening to the recording. During that time any parts of the transcription that did not make sense were clarified by moving back and forth on the recording. During the second reading, the researcher listened in detail, immersed herself in the words of the participant. The transcript was analysed line by line, to identify three types of exploratory comments, namely; descriptive, linguistic and conceptual, while trying to make sense of each participant’s experiences and being consciously aware not to change original meaning. The researcher tried to interpret the exploratory comments. This is the double hermeneutics of IPA. Then the researcher found a connection among emergent themes. Abstraction was used for the clustering of themes, as the superordinate theme was identified. Some differences were identified in some clusters, indicating polarization. Each interview was transcribed and analysed separately before moving on to the next case. Finally, all the themes clustered together and commonalities, differences and individuality were identified.

## Results

The superordinate theme demonstrated teamwork elicited the following themes:

### Delegation of duties

Participants highlighted that there is a need for someone to assume the leadership role during emergencies. The person who assumes the leadership role should be able to do introspection for his or her capabilities. As team leaders, the five participants all felt responsible to assign duties to other team members. Assigning duties in an emergency are important so as to ensure that tasks are carried out effectively. The effectiveness of assigned tasks needs to be confirmed through continuous mutual updates from team members. In a midwifery unit, every midwife should be competent to assume a leadership role and to direct team members in order to manage the case effectively, at the correct time and in the correct sequence, as affirmed by the following comments:

*‘I delegated duties to other team members and asked for the updates to ensure that duties are carried out and whether they are effective.’ [Thembi].*

*‘As the person who arrived first and assumed the leadership role, I felt I should assign duties to other team members such as taking vital signs, inserting intravenous cannulas and calculating the amount of blood loss.’ [Anga].*

‘*You have to delegate specific duties to your team members. If this person is responsible for observations and you want an equipment, you can’t ask this person to take an equipment for you.*’ *[Lunga].*

*‘Delegating duties to other team members ensures the duties are carried out. Specific duties were delegated to team members and team members kept me updated on the progress of the woman.’ [Zandie].*

### Importance of teamwork

Some of the participants were not aware that obstetric emergencies require a team effort. Participants acknowledged that the team leader should give out instructions and team members should be co-operative. Some participants felt that working in cooperation with other team members contributed towards the well-being of the woman. Also, most participants acknowledged the importance of teamwork in resolving obstetric emergencies. In a midwifery unit, collective teamwork results in better outcomes. Teamwork is alluded to by the following comments:

*‘Firstly, I didn’t know that when you are managing PPH you need more people. I knew that you call for help and you do some of the things then the person will come and help you.’ [Lunga].*

*‘I felt confident because I was able to do it with the help of my team members and it made me value teamwork more. This made me realise that there are certain things you cannot do alone.’ [Thembi].*

*‘Teamwork was very good because my team had knowledge about PPH so there was a good teamwork.’ [Sino].*

*‘Working with a team that wants to help and that keeps on advising made my job easy. I was a team leader who was unsure of what I was doing, but I knew that my team knows what to do.’ [Zandie].*

*‘Teamwork … I could say that my colleagues cooperated very well with me. They did everything I told them to do and then we managed the woman well.’ [Anga].*

### Team support

Having a supportive team helped the participants rely on fellow team members. The team members were not just a source of support but also help each other with critical thinking and decision making. Team members give advice when necessary, pointing out to the team leader when important steps are missed. In an emergency, every team member can have a positive contribution that could be of great benefit to the team and the emergency. One participant reported that her team demonstrated a sense of serenity and respect, as they were consciously aware that they were having a discussion about the woman and that the woman was present; therefore, they have to be careful speaking negatively about the process and the woman. The team leader needs to acknowledge and value other team members. Team support in the midwifery unit is a significant factor to pay attention to, to ensure positive midwifery outcomes. In a supportive midwifery unit, achievements are not regarded as belonging to any individual but to the whole unit. Team members who work in collaboration with one another avoid the chaos that defines many emergencies, as affirmed by the following comments:

*‘I just needed someone to reassure me that this is the step that follows after the other one. That’s how I did my critical thinking, and with the assistance of others.’ [Zandie].*

*‘The team leader was the one giving the instruction but others were just talking because it’s an emergency. You can’t keep quiet when you see that the team leader is missing something.’ [Lunga].*

*‘If another member has forgotten something, the team leader or another colleague will say please do this, not in a harsh way, but in a respectful way, so that even the woman does not sense that something is wrong.’ [Sino].*

### Confident team leader

Participants had varying experiences of the role of a team leader during the simulation. Being able to confidently delegate tasks and making sure that people carried out those tasks increased the student midwives’ confidence in being a leader. Participants reported seeing themselves as leaders in the real-life clinical situation. Simply being chosen to be a leader by one’s peers has the effect of increasing confidence. Knowing that people see the potential in one to lead has a motivating effect and encourage people to give in their best. Participants reported feeling nervous in the role but found that as they were immersed in the simulation, their capabilities and confidence increases. Assuming a leadership role in a simulation enables the participant to visualise a similar role for her/himself in the clinical setting. However, a lack of knowledge in a team leader about the subject matter can at times make the leader doubt his capabilities. The sense that team members had to take over at certain points made one participant feel less of a leader:

*‘I was not impressed because the things didn’t according to plan, so I’m not happy about being a leader …Yes, I did the job, but I missed some of the important points during the procedure so you can’t be impressed having your team members talking over you because you missed some important points whereas it’s your job.’ [Lunga].*

Others were more positive:

*‘The whole scenario, it’s challenging and when something is challenging, it brings out the best in you. I could see myself delegating to my peers and seeing them carrying out duties without complaints.*’ *[Thembi].*

*‘The fact that they chose me to be the team leader like they have trust and confidence in me that I can to do this, it means I can do this.’ [Zandie].*

*‘At first, I was nervous being chosen as a team leader, but I thought OK let me try it, but as time went on I felt proud of myself that I’m able to assign duties to people.’ [Anga].*

*‘I feel like I’m competent, even if I can come across PPH in the institution I will be able to take part in saving the woman’s life. You gain confidence and more experience when you do things practically.*

## Discussion

The present study was undertaken to explore the views of student midwives concerning teamwork during medium-fidelity obstetric emergency simulation. The team working during obstetric emergency provides good outcomes for both the woman and her baby. Most of the participants in this study were confident in delegating duties to team members. The role of the team leader during the emergency is of significance as he/she is the one who gives direction to team members. These findings were consistent with the findings of the studies conducted by Woods; Simkins; Gravlin [27–29] which reported that the participants were comfortable delegating duties. However, the findings of this study were in contrast with the findings of the study conducted by Johnson et al. [30] on Newly Qualified Nurses (NQN). The participants reported that Inadequate delegation skills resulted in an NQN who is stressed, anxious and unable to manage time adequately [30].

The participants of the present study reported that sharing duties among team members is important during emergencies. This is congruent with the findings of the study by Deering et al. [17], that monitoring the actions of others during teamwork ensures that the workload is evenly shared and woman safety is guaranteed. There is a need to clearly share and specify the duties to be performed by each member of the team in order to achieve the group goal. The participants of the present study affirmed that in order to ensure that the delegated work was carried out, the team leader should ask or be provided with regular updates by team members. Receiving updates from team members contributes to the leader’s awareness of the woman’s condition [17]. One participant felt that delegating people to perform specific functions is important so as to ensure that tasks are performed. However, Johnson et al. [30] point out that poor delegation by NQNs can result in loss of collaboration and a lack of a sense of responsibility by healthcare assistants (HCA).

The participants had good experiences working in a team; only one participant expressed a degree of ignorance about teamwork during obstetric emergencies. Monod et al. 22 found that simulated team training for the management of obstetric emergencies was considered useful. One participant acknowledged the importance of working in a team. Having a confident team ensures good outcomes. In a retrospective study by Siassakos et al. [31] on the effect of team training, good teamwork was deemed necessary for the management of obstetric emergencies. Poor teamwork is one of the causes of adverse events in woman care [2]. Working in close collaboration during emergencies is more likely to result in good outcomes for both the woman and the caregivers [32].

Two participants affirmed that co-operative teamwork brought good results and success. A sense that team members support and respect one another during obstetric emergencies is important. In this study, it was found that one participant recognised that team support enabled her to think better; to apply her critical thinking. Advice from team members during an emergency benefitted both the woman and the team leader. Phipps et al. [23] study on determining the implementation of labour and delivery team-training programmes with a simulation component stressed the importance of ‘shared responsibility’ and ‘cross monitoring’, which equipped team members to be assertive. Supporting each other during an emergency is done in a harmonious manner, demonstrating respect and good teamwork [17, 23]. Collective attributes of individual team members contribute to the performance of the team as a whole and tend to strengthen the confidence of the team leader. Participants had varied experiences of team leadership. One participant found the simulation challenging, thus bringing out the best in her. This is consistent with the findings of other studies, which found that it was the challenging aspect of simulation that enabled students to give of their best [33, 34]. Smith et al. [1] assert that simulation training in obstetric emergencies encourages teamwork and increases confidence in managing such emergencies. This is congruent with the experience of one participant, who felt that having a good team alongside her gave her the confidence to be a good team leader.

One participant felt nervous as a team leader initially but gained confidence as she became immersed in the leader’s role. Deering et al. [17] state that the role of the leader in an obstetrical emergency is to ensure that duties are performed effectively without unnecessary delays. Providing the students with an opportunity to practise a skill in a simulated environment increases students’ confidence in managing complications [35]. One participant felt that his limited knowledge inhibited his ability as a team leader. This is inconsistent with the other studies [36, 37] which found that the experiential learning pedagogy made possible in high-fidelity simulation (HFS) and root cause analysis facilitated student learning.

By creating a safe learning environment where students are allowed to make mistakes without causing harm to the patient, MFS has the effect of increasing student confidence. This safe learning environment can also be achieved through the use of teaching modalities such as high-fidelity simulation and root cause analysis [25, 26]. The participants asserted that the safe learning environment helped them to learn the necessary skills.

### Limitations

Given that the study focused on the lived experiences of fourth-year student midwives utilising simulation laboratories at one university, its findings cannot be generalized to other universities in South Africa. Therefore, exploring the views of the nursing students in other universities is needed.

## Conclusion

The participants gained confidence as they assumed leadership roles during the obstetric emergency simulation. The simulation provided the participants with an opportunity to exercise their delegation roles by assigning duties to team members. Student midwives had an opportunity to assume leadership roles and to function as part of a team in resolving an obstetric emergency. Medium–fidelity simulation provided a safe environment where collaborative teamwork can be practised.

## Abbreviations

ESMOE: Essential Steps in Management of Obstetrical Emergency
HCA: Health Care Worker
HFS: High-fidelity simulation
IPA: Interpretative phenomenological analysis
MFS: Medium-Fidelity Simulation
MOET: Managing Obstetrical Emergencies and Trauma
MOSES: Multidisciplinary Obstetric-Simulated Emergency Scenarios
NQN: Newly Qualified Nurse
PPH: Post-partum haemorrhage
PROMPT: Practical Obstetric Multi-Professional Training
UFH: University of Fort Hare
